# Bacteriological Evaluation of Gingival Crevicular Fluid in Teeth Restored Using Fixed Dental Prostheses: An In Vivo Study

**DOI:** 10.3390/ijms22115463

**Published:** 2021-05-22

**Authors:** Artak Heboyan, Mikayel Manrikyan, Muhammad Sohail Zafar, Dinesh Rokaya, Ruzan Nushikyan, Izabella Vardanyan, Anna Vardanyan, Zohaib Khurshid

**Affiliations:** 1 Department of Prosthodontics, Faculty of Stomatology, Yerevan State Medical University, Str. Koryun 2, Yerevan 0025, Armenia; annavardanyan@yahoo.co.uk; 2 Department of Pediatric Dentistry and Orthodontics, Faculty of Stomatology, Yerevan State Medical University, Str. Koryun 2, Yerevan 0025, Armenia; mikaelA@mail.ru (M.M.); vardnune@mail.ru (I.V.); 3 Department of Restorative Dentistry, College of Dentistry, Taibah University, Al Madinah, Al Munawwarah 41311, Saudi Arabia; MZAFAR@taibahu.edu.sa; 4 Department of Dental Materials, Islamic International Dental College, Riphah International University, Islamabad 44000, Pakistan; 5 Department of Clinical Dentistry, Walailak University International College of Dentistry, Walailak University, Bangkok 10400, Thailand; 6 Davidyants Laboratories, Department of Microbiology, GYSANE Limited Liability Company, Yerevan 0054, Armenia; doctorruz1@gmail.com; 7 Department of Prosthodontics and Implantology, College of Dentistry, King Faisal University, Al-Hofuf, Al-Ahsa 31982, Saudi Arabia; drzohaibkhurshid@gmail.com

**Keywords:** biomaterials, molecular biology, metal-ceramic, zirconium, CAD/CAM, crowns, gingival pocket, periodontium, dental biofilm, oral microflora, oral microorganisms

## Abstract

The present in vivo study determined the microbiological counts of the gingival crevicular fluid (GCF) among patients with fixed dental prostheses fabricated using three different techniques. A total of 129 subjects were divided into three study groups: first, cobalt-chrome-based, metal-ceramic prostheses fabricated by the conventional method (MC, *n* = 35); the second group consisted of cobalt-chrome-based, metal-ceramic prostheses fabricated by the computer-aided design and computer-aided manufacturing (CAD/CAM) technique (CC-MC, *n* = 35); the third group comprised zirconia-based ceramic prostheses fabricated using the CAD/CAM technique (CC-Zr, *n* = 35). The control consisted of 24 patients using prostheses fabricated with either MC, CC-MC, or CC-Zr. The GCF was obtained from the subjects before treatment, and 6 and 12 months after the prosthetic treatment. Bacteriological and bacterioscopic analysis of the GCF was performed to analyze the patients’ GCF. The data were analyzed using SPSS V20 (IBM Company, Chicago, IL, USA). The number of microorganisms of the gingival crevicular fluid in all groups at 12 months of prosthetic treatment reduced dramatically compared with the data obtained before prosthetic treatment. Inflammatory processes in the periodontium occurred slowly in the case of zirconium oxide-based ceramic constructions due to their biocompatibility with the mucous membranes and tissues of the oral cavity as well as a reduced risk of dental biofilm formation. This should be considered by dentists and prosthodontists when choosing restoration materials for subjects with periodontal pathology.

## 1. Introduction

Fixed dental prostheses have been clinically determined to play important roles in the restoration of teeth or replacement of missing teeth [1,2,3]. Prosthetic rehabilitation using fixed dental prostheses improves the quality of life and oral health of the patients. Dental prostheses can be of full metal, metal veneered with ceramic (metal-ceramic), or full ceramic zirconia-based ceramics fabricated by the computer-aided design and computer-aided manufacturing (CAD/CAM) technology [3,4]. The life span of fixed dental prostheses depends on the condition of the periodontium in abutment teeth because the mucous membranes in this area are subject to constant mechanical trauma and microbial challenges [5,6].

Various oral microbial species present in the biofilm attached to oral tissues and materials are mainly associated with the inflammatory reaction in the oral tissues. The extent and severity of inflammation vary depending on the properties of the biomaterials used for prosthodontic constructions and the nature of the oral microbial species present in the biofilm [7,8]. The pathological bacterial species found in various oral diseases are also present in normal oral flora, having complex interactions with the dynamic oral environment and other bacterial species. In general, microorganisms are symbionts, and in health status, the oral microbiome sustains a high degree of homeostasis [9].

The majority of oral microorganisms are commensal and do not cause any harm to the host. Although the species diversity of microbial biocenosis in different parts of the human body alters regularly, each individual characteristically presents more or less peculiar microbial communities. According to Bergey’s classification, the closely related families *Bacteriadaseae, Porphyromonadaceae*, and *Prevotellaceae* comprise the *Bacteroidetes* type [10]. The difficulties in the bacteriologic distinguishing of *Bacteroid* cultures in normal and in various pathologic conditions of the oral cavity do not allow for identifying a definite causative agent of the disease. Gingival crevicular fluid (GCF) is a body fluid specific to the gingival crevice and is an indicator of periodontal health, and its analysis may help in diagnosing oral diseases [11,12,13]. The GCF can be collected noninvasively using a cost-effective and site-specific collection strategy and is an ideal tool to detect host–bacterial interactions and to reflect the severity of periodontal inflammation originating from host cells and the numerous microbes harbored in inflamed periodontal pockets from dental prostheses [14,15,16].

Periodontal disease is an inflammatory condition of the periodontal tissues supporting the teeth (gingiva, cementum, periodontal ligament, and alveolar bone) and is widespread in adult populations [14]. The differential microorganisms and metabolites in GCF between patients with periodontitis and healthy individuals are possible biomarkers, pointing to a potential strategy to predict, diagnose, prognose, and manage personalized periodontal therapy [17]. Periodontal disease is associated with imbalanced immune homeostasis in the oral mucosa, with increased bacterial growth, and multiplication of the dental plaque on the prostheses. However, insufficient data is available on periodontal pathogens in relation to periodontal disease and in individuals with healthy periodontium. In addition, the relationship between host–bacterial interactions and biochemical metabolism has not been clearly identified [18,19].

The degree of biofilm formation on various dental ceramics depends on the species of microorganisms [20]. Restorative biomaterials may influence the formation of biofilm because high surface energy and rough and irregular surfaces create a favorable environment for bacterial colonization. However, ideal material characteristics [21] and fabrication methods remain insufficiently investigated. Furthermore, no sufficient data are available on changes in the microbiological parameters of GCF in prosthodontic treatment with fixed dental constructions made by various fabrication techniques. The influence of resident microorganisms of the oral cavity on the localization of pathological processes is not fully understood and requires further investigation. Thus, the present study aimed to determine the microbiological composition and counts of the GCF among patients with fixed dental prostheses fabricated using various biomaterials and fabrication techniques.

## 2. Results

There was no significant difference in terms of demographic characteristics (age and gender) of the study participants of study groups (Table S1). In all, 35 MC, 35 CC-MC, and 35 CC-Zr prostheses had gingivitis and/or periodontitis, and 24 prostheses fabricated either from MC, CC-MC, or CC-Zr were healthy (control) (Table 1).

The microbiological composition of the GCF in all groups before treatment is presented in Table 2. The GCF in subjects with healthy gums and those with gingivitis and/or periodontitis before prosthetic treatment showed the quantitative prevalence of microorganisms such as *Veilonella* spp., *Corynebacterium anaerobium*, and *Neisseria* spp. in the MC group; *Streptococcus haemolyticus (β-haem. Str.)*, *Str. spp. viridans*, *Neisseria* spp., and *Veilonella* spp. in the CC-MC group; and *Str. spp viridans*, Staphylococcus aureus (*St. aureus*), and *Corynebacterium anaerobium* in the CC-Zr group. *Veillonela* spp. was absent in the CC-MC group. *Veillonela* spp. was observed in a greater number in the healthy and CC-Zr groups, and *Beta-hemolytic streptococcus* was observed in a greater number in the MC and CC-MC groups.

The multiple comparisons of the microbiological compositions and counts of the GCF showed that *Neisseria* spp. significantly differed between the healthy vs. CC-MC groups (*p* = 0.041), and *Staphylococcus* spp. significantly differed between the healthy vs. CC-Zr groups (*p* = 0.004), the MC vs. CC-Zr groups (*p* = 0.002), and the CC-MC vs. CC-Zr groups (*p* = 0.023). *Staphylococcus* spp. and *Corynebacterium* spp. significantly differed between the CC-MC vs. CC-Zr groups (*p* = 0.015) (Table S2).

**Figure 1:**
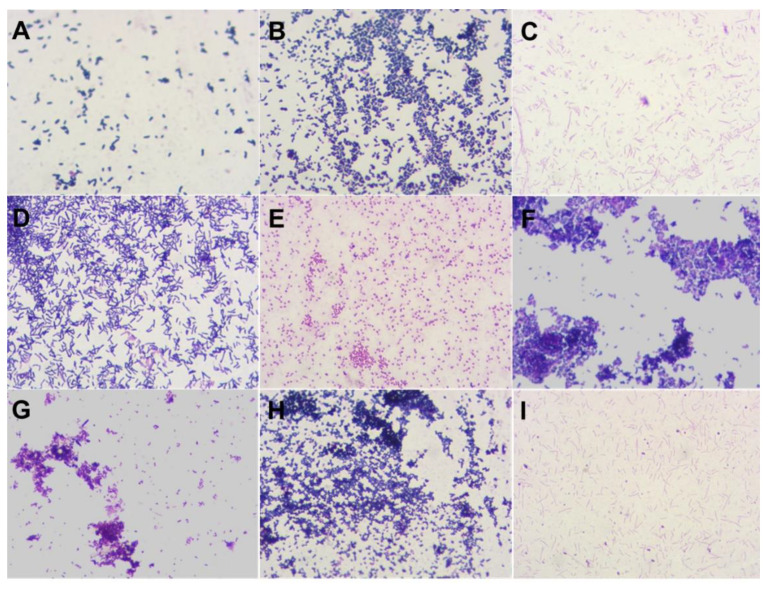
Microorganisms observed in the GCF: *Lactobacillus* spp. (light microscopy, magnification × 1000, Gram stain) (**A**), *Streptococcus* spp. (light microscopy, magnification × 1000, Gram stain) (**B**), *Fuzobacterium* spp. (light microscopy, magnification × 1000, Gram stain) (**C**), *Corynebacterium* spp. (light microscopy, magnification × 1000, Gram stain) (**D**), *Neisseria* spp. (light microscopy, magnification × 1000, Gram stain) (**E**), *Peptocococcus* spp. (light microscopy, magnification × 1000, Gram stain) (**F**), *Peptocococcus* spp., *Peptostreptococcus* spp. (light microscopy, magnification × 1000, Gram stain) (**G**), *Staphylococcus* spp., *Streptococcus* spp. (light microscopy, magnification × 1000, Gram stain) (**H**), *Fuzobacterium* spp.—isolated *streptococci* are visible in the field of view (light microscopy, magnification × 1000, Gram stain) (**I**).

Figure 1 shows the microbiology figures of various organisms: *Lactobacillus.* spp. (Figure 1A), *Streptococcus* spp. (Figure 1B), *Fuzobacterium* spp. (Figure 1C), *Corynebacterium* spp. (Figure 1D), *Neisseria* spp. (Figure 1E), *Peptocococcus* spp. (Figure 1F), *Peptocococcus* spp., *Peptostreptococcus* spp. (G), *Staphylococcus spp., Streptococcus* spp. (Figure 1H), *Fuzobacterium spp.*—isolated *streptococci* are visible in the field of view (Figure 1I).

The microbiological compositions of the GCF in all groups at six months of prosthetic treatment are presented in Table 3. *Peptostreptococcus* spp. and *Beta-hemolytic streptococcus* were seen in greater numbers in the MC group. *Veillonela* spp. was observed in the MC, CC-MC, and CC-Zr groups.

The multiple comparisons of microbiological compositions and counts of the GCF showed that *Beta-hemolytic streptococcus* significantly differed between the healthy vs. MC (*p* = 0.025) and between the MC vs. CC-MC groups (*p* = 0.024) (Table S2).

The microbiologic compositions of the GCF in all groups at 12 months of prosthetic treatment are presented in Table 4. *Peptococcus* spp. was observed in greater numbers in the healthy, MC, and CC-Zr groups. It was shown that the number of microorganisms dramatically reduced in all the groups compared with the data obtained before prosthetic treatment. Moreover, *C. anaerobium* was absent in the healthy group after one year of prosthetic treatment.

The multiple comparisons of the microbiologic compositions and counts of the GCF showed that *Peptococcus* spp. significantly differed between the CC-MC vs. CC-Zr groups (*p* = 0.025), and *Beta-hemolytic streptococcus* significantly differed between the healthy vs. MC (*p* = 0.001), between the MC vs. CC-MC (*p* = 0.002), and between the MC vs. CC-Zr groups (*p* = 0.002) (Table S2).

## 3. Discussion

The present study investigated the oral microflora and microbiological compositions of the GCF among patients using fixed dental prostheses fabricated by various biomaterials and fabrication techniques. The present study reported the presence of pathogenic microbial composition in all the observation groups. The observed clinical picture was diverse in fixed prosthodontics and showed peculiarities depending on the type of biomaterial and technique used for fabrication. All the patients examined were healthy and reported no medical history. Pronounced growth of microorganism colonies was observed in all groups before treatment. Among the periodontal pathogenic bacteria, *Porphyromonas gingivalis (P. Gingivalis)* exhibited the unique ability to coaggregate not only with *Fuzobacterium* spp. but also with early colonizers, such as *Streptococcus* spp. [22,23]. This explains its early occurrence in developing dental biofilm [24], which is frequently discharged from the deep periodontal pockets among adult patients with periodontitis. Virulence of *P. Gingivalis* is associated with an increase of cytokines released by the protective host cells [25,26]. Even being a minor component of subgingival microbiota, it significantly affects the ecosystem, destroying the innate immunity pathways. Gram-negative anaerobe bacteria (such as *F. nucleatum*) play an important role in biofilm maturation, acting as a link between early and late colonizers and directing the architecture of biofilm, and therefore, improving the adhesion of more bacteria associated with periodontitis [27,28]. Similar to *P. gingivalis*, *F. nucleatum* is also able to join and penetrate the host epithelial cells and stimulate a host immune inflammatory reaction. Manifesting in association with other microorganisms, *Peptostreptococci* are usually pathogens of mixed infections.

During our research, at six months of prosthetic treatment, the aggravation of indicators was noted among patients with gingivitis in the MC group. The amount of *Fuzobacterium* spp., *Streptococcus haemolyticus*, *St. aureus*, and *Viridans streptococci* increased, probably due to the exacerbation of the process because of tooth preparation and noncompliance with individual hygiene. Inflammatory purulent processes occurring with the participation of associations, consisting of *Peptococcus* spp. and *Peptostreptococcus* spp. are more severe and extensive than lesions caused by the monoculture of *anaerobic gram-positive cocci*, because *β-hemolytic streptococci* are pyogenic and pathogenic to humans, and *St. aureus* is the cause of purulent bacterial and generalized infections [9].

Various degrees of contamination of gingival sulcus with microorganisms were noted at different fixed prosthodontic constructions. *Candida albicans* type of fungi revealed in smears before the treatment further resulted in the inflammatory process in periodontal tissues when using metal-ceramic constructions of the conventional manufacturing method, which we associated with the weakening of the immune defense in the gingival sulcus. A similar picture of the quantitative difference in microflora composition was observed when fixed metal-ceramic constructions were fabricated using the CAD/CAM technology. Compared with patients of other groups, the best results both in quantitative and qualitative composition of microflora in gingival sulcus were achieved when using zirconia-based fixed constructions fabricated using CAD/CAM technology. Sanitation of the oral cavity resulted in significantly decreased contamination of the periodontal sulcus in the MC and CC-MC observation groups 12 months after prosthodontics (Table 4). The amount of *Prevotella intermedia*, *Porphyromonas gingivalis, Streptococcus haemolyticus, Fuzobacterium* spp., and *Corynebacterium anaerobium* among the patients of all observation groups reduced dramatically after 12 months of prosthodontic treatment.

Notably, *Candida albicans* type of fungi was found both during bacteriologic and bacterioscopic research in all groups, regardless of the duration of the study, which reliably confirmed their presence. However, it should also be present normally in the form of saprophytes. Thus, quantitative and qualitative pathology of fungal elements can be revealed only by bacterioscopy. We mentioned the presence of pathology in detecting more than six well-stained *pseudomycelia*. Fungi were found in two patients with gingivitis in the MC and CC-MC observation groups, and only in one patient in the CC-Zr group. Among the patients with periodontitis, fungi were found among five patients (18.5%) of the MC group; two patients (12.5%) of the CC-MC group, and one patient (6.7%) of the CC-Zr group 12 months after prosthetic treatment.

Summing up the results of the clinical and laboratory research methods using various construction techniques for prosthodontic treatment, it could be argued that clinical deterioration of the gums due to insufficient marginal fit created a potential retentive area and promoted the adhesion of the dental biofilm that occurs following prosthodontic rehabilitation using the conventional metal/ceramic constructions. A negative effect of metal-ceramic prostheses on the denture bearing area, leading to gingivitis and periodontitis, is associated not only with mechanical damage to the gums during tooth preparation but also with irregular outlines and topography of the crown edge, with the formation of dental biofilm along this edge. Thus, a faulty marginal fit of the crown to the neck of the tooth could cause marginal leakage, contributing to the destruction of the adhesive cement layer and the penetration of microorganisms [29,30].

In all three groups, mechanical preparation appeared to be traumatic and resulted in the deterioration of periodontal tissues. However, 12 months later, we observed periodontal healing among subjects whose constructions had used CAD/CAM technology. In addition, a more pronounced shift towards clinical recovery was observed in the group with zirconia-based constructions because zirconium is less aggressive to periodontal tissues and has fewer negative effects on the gingival margins. In this type of prosthesis, inflammatory processes in periodontium do not occur, quickly improving the marginal fit and reducing the risk of formation of dental biofilm. In addition, zirconium dioxide is biocompatible with the mucous membranes and tissues of the oral cavity [8,29].

Thus, the condition of periodontal tissues was slightly better in cases where prosthetics were carried out with constructions made using CAD/CAM technology, as shown in the results of the study. This could have been due to the material rather than the processing method [31,32].

The CAD/CAM fabricated prostheses exhibited a better periodontal response. Pabst et al. [32] analyzed various CAD/CAM ceramic biomaterials (e.max CAD HT, e.max CAD LT, Mark II, and Empress CAD) on cell viability, oral keratinocytes (HOK), adenylate kinase (ADK), and secretion of human gingival fibroblasts. The CAD/CAM fabricated materials showed significant variations in cell viability and migration ability of HGF. Similarly, Shang et al. [31] investigated the association of tumor necrosis factor-α and interleukin-6 with CAD/CAM zirconia and conventional Ni-Cr metal-ceramic prostheses. The volume of GCF, tumor necrosis factor-α, interleukin-6, sulcus bleeding index, and probing depth were significantly increased in conventional Ni-Cr metal-ceramic prostheses (*p* > 0.05).

Heboyan et al. [8] studied inflammation dynamics using cytomorphometric analysis of the periodontium before and after the use of fixed dental prostheses using the conventional method (C/M/CoCr), cobalt-chrome metal-ceramic prostheses using the CAD/CAM technique (C/C/CoCr), and zirconia-based ceramic prostheses using the CAD/CAM technique (C/C/Zr) among subjects with gingivitis and periodontitis. They found that regardless of prostheses type used, no significant change in the parameters was identified among patients with a healthy periodontium, before and after prosthetic treatment. In all study groups, oral epithelial cell counts significantly increase (*p*-value < 0.05) while the polymorphonuclear neutrophils count significantly decreased (*p*-value < 0.05) following the use of the fixed prostheses. However, in this present study, the CAD/CAM fabricated prostheses exhibited a better periodontal response.

Recently, Avetisyan et al. [33] studied the role of different kinds of fixed constructions on the periodontium and investigated the relationship of gum recession and gingival biotype to prosthesis types. Patients with CAD/CAM fabricated restorations demonstrated better periodontal outcomes compared with those with conventional metal-ceramic construction. Moreover, zirconia-based restorations showed improved periodontal conditions, reduced inflammation, and conservation of oral hygiene. The authors concluded that the specific gingival biotype before prosthetic treatment should be considered to avoid trauma to the periodontium and inhibit the occupation of microbes.

Various new techniques have been used to access periodontal disease in fixed dental prostheses. Pei et al. [17] studied the oral microbiome, the oral metabolome, and the link between them, and identified potential molecules as useful biomarkers for predictive, preventive, and personalized medicine in generalized chronic periodontitis. They found that the microorganisms, metabolites in GCF, and clinical data combined showed a clear trend, and clinical data regarding periodontitis could be reflected in the shift of the oral microbial community and the change in metabolites in GCF. Combined citramalic acid and N-carbamylglutamate yielded satisfactory accuracy (AUC  =  0.876) for the predictive diagnosis of generalized chronic periodontitis. Similarly, Sinjari et al. [2] studied volatile sulfur compounds (VSCs) using Oral Chroma™ among patients wearing provisional and permanent fixed prostheses, who were treated or not with supportive nonsurgical periodontal therapy. They found that Oral Chroma™ produced a comprehensive assessment of VSC in the clinical diagnosis of halitosis and that professional oral hygiene seemed to influence VSC production. In the present study, we did not study these sulfur compounds.

The present study encountered a few limitations. Due to the time factor, we could not enlarge the sample size. The present study did not evaluate the association of gingivitis and periodontitis with different factors related to fixed prostheses, including margin placement, individual variations, and pontic design, which may have influenced the outcome [34]. Due to a smaller sample size, we could not further divide the control group participants based on the prosthesis type, therefore restricting a direct comparison of the periodontitis group prosthesis group with the respective controls. Future studies should focus on investigating the correlation of periodontitis stages, prosthetic factors (margin design, occlusal status, and pontic design) for a prolonged period using a larger sample size. The current research determined that prosthodontic management of periodontitis patients using the CAD/CAM technique (CC-MC and CC-Zr) improved the microbiologic outcomes compared with those of the conventional fixed prostheses.

## 4. Materials and Methods

### 4.1. Ethics Approval

This research was carried out in Nord KS Dental Clinics, and laboratory analyses were performed at Davidyants Laboratories (GYSANE Limited Liability Company, Yerevan, Armenia) from August 2016 to June 2019. This study was approved by the Institutional Ethics Committee of the Yerevan State Medical University (IRB approval N12-5/2019). The study details were explained to each participant according to the Declaration of Helsinki. Before initiating treatment, all the study participants signed written consent.

### 4.2. Patient Selection Criteria

The minimum sample size was calculated using G*Power Software (V 3.1.9.7, Heinrich-Heine-Universität Düsseldorf, Germany) and following parameters (effect size = 0.4; ɑ-error = 0.05; power of the test = 0.95). The power analysis of the corresponding sample size was performed using a power value of 0.95 and was based on the assumptions of normality and approximate common variance among the study groups. Accordingly, a total of 129 subjects were selected who met the criteria on a voluntary basis. Inclusion criteria included healthy subjects with no past medical history or systemic diseases, and patients not receiving any medications within the past six months. Exclusion criteria included breastfeeding or pregnant women, smokers, and subjects who underwent surgical and/or nonsurgical periodontal therapy. Subjects with gingivitis or periodontitis and healthy periodontium subjects who needed prosthetic treatment with full coverage fixed prosthetic constructions were selected.

### 4.3. Study Groups

The present study included a total of 129 patients/prostheses (one prosthesis was fabricated for each patient), divided into three groups; cobalt-chromium-based metal-ceramic constructions fabricated by the conventional technique (MC), cobalt-chromium-based metal-ceramic constructions fabricated using CAD/CAM technology (CC-MC), and zirconia-based ceramics fabricated by CAD/CAM technology (CC-Zr). The gingivitis and/or periodontitis group (*n* = 105) consisted of 35 prostheses of each type, including MC, CC-MC, and CC-Zr. The control group included patients (*n* = 24) who were in a good state of general and periodontal health, using prostheses fabricated either from MC, CC-MC, or CC-Zr.

### 4.4. Prostheses Fabrication

Three types of prostheses were fabricated: MC, CC-MC, and CC-Zr. For the MC prostheses, at first, the initial wax copings were replaced by metal copings using the lost-wax technique, followed by bonding the ceramic layer (Vita Vm9; Vita Zahnfabrik, Bad Sackingen, Germany) on the metallic coping. The CC-MC prostheses were fabricated using CAD/CAM technology by Ceramill Sintron Technology (Amann Girrbach AG, Austria). Briefly, the copings were milled from soft pre-sintered cobalt-chromium alloy (Ceramill Sintron 71 XS; Amann Girrbach AG, Austria)by CAD/CAM and then sintered under high pressure. For the CC-Zr prostheses, the zirconia-based ceramics cores were milled by CAD/CAM technology from pre-sintered zirconia blocks (Zolid; Amann Girrbach AG, Austria). These prostheses were then sintered, and porcelain layers were applied. The final prostheses were tested, fitted on their respective casts, and glazed.

### 4.5. Laboratory Analysis

Bacteriological and bacterioscopic research methods were used to examine the patients’ GCF. The GCF from the gingival crevice/periodontal pocket was collected before treatment, and 6 and 12 months after prosthetic treatment from subjects with gingivitis or periodontitis, and subjects without periodontal pathology (healthy). For this purpose, the area was isolated by cotton rolls and dried by oil-free air spray. A sterile absorbent paper strip (Periopaper, Oraflow Inc., Smithtown, NY, USA) was inserted in the gingival crevice using forceps. The Periopaper was inserted in the bottom of each gingival sulcus (~1 mm). After 30 s of maintenance in the sulcus, the Periopaper was removed. The paper strips were stored in vials and transported to the laboratory using Hicultur Transport swab Himedia in Amies Charcoal medium (Thermo Fisher Scientific, Invitrogen BioServices, Mumbai, India) [8,29].

Microbiological cultures under aerobic, micro-aerophilic, and anaerobic conditions were carried out using the following main nutrient media (all from Liofilchem, Italy): Columbia agar, Chocolate agar, Schaedler agar, Sabouraud CAF agar, Endo agar, Mannitol salt agar, and MRS agar. Cultivation of aerobic bacteria was carried out for 1 to 3 days at 37 °C, while fungi were cultivated at 30 °C. The cultures of microaerophiles and anaerobe bacteria were carried out on an aerostat produced by bioMerieux (bioMerieux, France). GENbox gasbags by bioMerieux (bioMerieux, France) were used in the aerostat for micro-aerophile and anaerobe bacteria. The incubation period was 2 to 3 days for microaerophilic bacteria and 4 to 7 for strict anaerobes at a temperature of 37 °C. The anaerobic condition was controlled using an anaerobic indicator by bioMerieux (bioMerieux, France).

### 4.6. Statistical Analysis

Statistical analysis of the results was performed using SPSS, Version 20 (IBM Company, Chicago, IL, USA). Descriptive statistics were calculated. One-way ANOVA with post hoc Sheffe was used to compare among the groups. The level of significance was set at *p* = 0.05.

## 5. Conclusions

Clinical deterioration of the gums occurred at six months after prosthetics with conventional metal-ceramic constructions. The periodontal microbiological compositions and counts were slightly better in cases where prosthetics were carried out with constructions fabricated using CAD/CAM technology. A more pronounced shift towards clinical recovery was observed in the group with zirconia-based constructions used because zirconia is highly biocompatible to periodontal tissues and has fewer negative effects on gingival margins. Inflammatory processes in the periodontium did not occur promptly due to the zirconium oxide’s biocompatibility with the mucous membranes and tissues of the oral cavity that inhibited the formation of dental biofilm. This should be considered by dentists and prosthodontists while counseling patients concerning oral hygiene, as well as motivating people regarding periodontal pathology to choose CAD/CAM fabricated zirconium oxide-based constructions, which have fewer negative effects on the periodontium.

## Figures and Tables

**Table 1:** Distribution of subjects in three groups according to their periodontal status.

Subject Details	Details
Mean age of the subjects	34 years old (18–50) years old
Total Subjects/Prostheses With healthy gums (MC, CC-MC, and CC-Zr) With gingivitis and/or periodontitis	129
	24
	105
MC CC-MC CC-Zr	35 35 35

MC = Cobalt-chrome-based, metal-ceramic prosthesis fabricated by conventional method; CC-MC = metal-ceramic prostheses fabricated using computer-aided design and computer-aided manufacturing (CAD/CAM) technique; CC-Zr = CAD/CAM fabricated zirconia-based ceramic prostheses.

**Table 2:** Microbiologic composition and counts of the GCF in all groups before treatment.

Microorganism	Healthy (CFU/s)		MC (CFU/s)		CC/MC (CFU/s)		CC/Zr (CFU/s)	
	Mean	SD	Mean	SD	Mean	SD	Mean	SD
*Enterococcus* spp.	6.297 × 10^4^	2.037 × 10^5^	1.601 × 10^5^	3.554 × 10^5^	1.057 × 10^5^	2.724 × 10^5^	2.323 × 10^5^	4.261 × 10^5^
*Peptostreptococcus* spp.	5.137 × 10^5^	2.031 × 10^6^	7.576 × 10^5^	2.365 × 10^6^	9.300 × 10^5^	2.742 × 10^6^	2.259 × 10^5^	3.759 × 10^5^
*Neisseria* spp. *^§^*	2.597 × 10^4^	4.374 × 10^4^	5.369 × 10^4^	1.725 × 10^5^	2.253 × 10^5^	3.974 × 10^5^	6.241 × 10^5^	2.121 × 10^5^
*Peptococcus* spp.	2.242 × 10^5^	4.077 × 10^5^	1.913 × 10^5^	3.812 × 10^5^	5.861 × 10^5^	1.994 × 10^6^	2.446 × 10^5^	4.205 × 10^5^
*Staphylococcus* spp. *^ψ^* *^δ^*	1.821 × 10^4^	3.749 × 10^4^	1.968 × 10^4^	3.791 × 10^4^	6.184 × 10^4^	1.994 × 10^4^	2.742 × 10^5^	4.548 × 10^5^
*Beta-hemolytic streptococcus*	1.045 × 10^2^	2.787 × 10^2^	1.247 × 10^6^	3.252 × 10^6^	1.621 × 10^6^	3.732 × 10^6^	1.263 × 10^4^	2.861 × 10^4^
*Candida albicans*	1.708 × 10^1^	3.793 × 10^1^	3.655 × 10^3^	1.718 × 10^4^	1.292 × 10^2^	3.293 × 10^3^	1.595 × 10^2^	3.443 × 10^2^
*Alpha-haemolytic streptococcus*	1.184 × 10^3^	2.753 × 10^3^	1.368 × 10^5^	3.221 × 10^5^	1.644 × 10^6^	3.726 × 10^6^	6.023 × 10^5^	2.127 ×10^6^
*Lactobacillus* spp.	5.779 × 10^5^	2.033 × 10^6^	7.173 × 10^5^	2.371 × 10^6^	4.656 × 10^5^	1.996 × 10^6^	1.056 × 10^5^	2.915 × 10^5^
*Corynebacterium* spp. *^§^* *^δ^*	1.630 × 10^4^	3.264 × 10^4^	7.884 × 10^4^	2.367 × 10^5^	2.533 × 10^5^	4.294 × 10^5^	1.068 × 10^4^	2.911 ×10^4^
*Fusobacterium* spp.	5.100 × 10^3^	2.041 × 10^4^	6.363 × 10^4^	2.382 × 10^5^	4.164 × 10^4^	1.996 × 10^5^	5.104 × 10^3^	2.130 × 10^4^
*Porphyromonas gingivalis*	1.054 × 10^3^	2.784 × 10^3^	1.352 × 10^4^	2.768 × 10^4^	6.376 × 10^3^	1.988 × 10^4^	6.161 × 10^5^	2.123 × 10^6^
*Prevotella intermedia*	1.058 × 10^3^	2.783 × 10^3^	6.662 × 10^3^	1.706 × 10^4^	3.120 × 10^3^	4.395× 10^3^	5.626 × 10^5^	2.127 × 10^6^
*Veillonella* spp.	6.520 × 10^6^	2.031 × 10^7^	1.804 × 10^8^	3.482 × 10^8^	.	.	4.382 × 10^7^	4.798 × 10^7^

*^§^* Significant difference between healthy vs. CC-MC; *^ψ^* Significant difference between healthy vs. CC-Zr; *^δ^* Significant difference between CC-MC vs. CC-Zr; Post hoc Scheffe. Significant at *p* < 0.05.

**Table 3:** Microbiologic composition and counts of the GCF in all groups at six months of prosthetic treatment.

Microorganisms	Healthy (CFU/s)		MC (CFU/s)		CC/-MC (CFU/s)		CC/Zr (CFU/s)	
	Mean	SD	Mean	SD	Mean	SD	Mean	SD
*Enterococcus* spp.	4.262 × 10^5^	2.039 × 10^3^	1.358 × 10^5^	3.225 × 10^5^	2.224 × 10^4^	3.986 × 10^4^	1.795 × 10^4^	3.367 × 10^4^
*Peptostreptococcus* spp.	5.212 × 10^5^	2.028 × 10^6^	1.235 × 10^6^	3.248 × 10^6^	1.596 × 10^4^	3.197 × 10^4^	1.075 × 10^5^	2.908 × 10^5^
*Neisseria* spp.	5.502 × 10^4^	2.040 × 10^5^	2.413 × 10^4^	4.278 × 10^4^	9.488 × 10^3^	2.742 × 10^4^	1.519 × 10^4^	3.465 × 10^4^
*Peptococcus* spp.	1.371 × 10^5^	3.342 × 10^5^	1.870 × 10^5^	3.826 × 10^5^	2.384 × 10^3^	3.910× 10^3^	1.390 × 10^5^	3.502 × 10^5^
*Staphylococcus* spp.	4.363 × 10^3^	2.037 × 10^4^	1.965 × 10^4^	3.792 × 10^4^	2.144 × 10^3^	4.021 × 10^3^	1.510 × 10^4^	3.469 × 10^4^
*Beta-hemolytic streptococcus ^§ η^*	9.583 × 10^1^	2.804 × 10^2^	1.829 × 10^6^	3.846 × 10^6^	1.424 × 10^4^	3.255 × 10^4^	1.236 × 10^3^	2.873 × 10^3^
*Candida albicans*	2.083 × 10^1^	4.148 × 10^1^	6.218 × 10^5^	2.385 × 10^6^	4.452 × 10^2^	2.001 × 10^3^	1.545 × 10^1^	3.460 × 10^1^
*Alpha-haemolytic streptococcus*	6.421 × 10^2^	2.033 × 10^3^	1.335 × 10^5^	3.226 × 10^5^	4.741 × 10^5^	1.994 × 10^6^	2.378 × 10^4^	4.240 × 10^4^
*Lactobacillus* spp.	1.980 × 10^4^	3.688 × 10^4^	7.319 × 10^4^	2.373 × 10^5^	4.893 × 10^4^	2.001 × 10^5^	9.278 × 10^4^	2.936 × 10^5^
*Corynebacterium* spp.	5.146 × 10^5^	2.039 × 10^6^	1.272 × 10^6^	3.247 × 10^6^	8.181 × 10^5^	2.763 × 10^6^	6.145 × 10^3^	2.124 × 10^4^
*Fusobacterium* spp.	9.637 × 10^3^	2.803 × 10^4^	1.357 × 10^4^	3.225 × 10^4^	1.260 × 10^3^	3.300 × 10^3^	2.772 × 10^2^	4.534 × 10^2^
*Porphyromonas gingivalis*	5.083 × 10^1^	2.041 × 10^2^	3.425 × 10^4^	1.715 × 10^5^	2.628 × 10^3^	4.244 × 10^3^	1.524 × 10^4^	3.463 × 10^4^
*Prevotella intermedia*	2.208 × 10^1^	4.096 × 10^1^	6.460 × 10^3^	2.374 × 10^4^	1.561 × 10^3^	3.209 × 10^3^	1.120 × 10^4^	2.898 × 10^4^
*Veillonella* spp.	7.825 × 10^5^	2.014 × 10^6^	4.886 × 10^7^	1.722 × 10^8^	1.117 × 10^7^	2.707 × 10^7^	9.860 × 10^7^	2.925 × 10^8^
*Corynebacterium* *anaerobium*	0.000	0.000	4.153 × 10^4^	1.724 × 10^5^	5.640 × 10^4^	2.001 × 10^5^	5.454 × 10^3^	2.132 × 10^4^

*^§^* Significant difference between healthy vs. CC-MC; *^η^* Significant difference between MC vs. CC-MC; Post hoc Scheffe. Significant at *p* < 0.05.

**Table 4:** Microbiologic composition and counts of the GCF in all groups at 12 months of treatment.

Microorganism	Healthy (CFU/s)		MC (CFU/s)		CC/MC (CFU/s)		CC/Zr (CFU/s)	
	Mean	SD	Mean	SD	Mean	SD	Mean	SD
*Enterococcus* spp.	1.291 ×10^2^	3.368 × 10^2^	1.651 × 10^4^	3.539 × 10^4^	1.496 × 10^3^	3.234 × 10^3^	8.422 × 10^3^	2.098 × 10^4^
*Peptostreptococcus* spp.	1.072 × 10^5^	2.777 × 10^5^	9.391 × 10^5^	2.861 × 10^6^	1.184 × 10^3^	2.692 × 10^3^	2.338 × 10^5^	4.257 × 10^5^
*Neisseria* spp.	4.725 × 10^3^	2.039 × 10^4^	1.037 × 10^4^	2.852 × 10^4^	9.208 × 10^3^	2.745 × 10^4^	2.746 × 10^4^	4.546 × 10^4^
*Peptococcus* spp. *^δ^*	2.133 × 10^5^	4.122 × 10^5^	4.385 × 10^4^	1.711 × 10^5^	2.064 × 10^3^	3.562 × 10^3^	2.778 × 10^5^	4.531 × 10^5^
*Staphylococcus* spp.	8.041 × 10^1^	2.016 × 10^2^	8.541 × 10^3^	2.356 × 10^4^	1.736 × 10^2^	3.695 × 10^2^	2.327 × 10^3^	4264.268 4.264 × 10^3^
*Beta-hemolytic streptococcus ^§ η ß^*	1.708 × 10^1^	3.793 × 10^1^	2.991 × 10^4^	4.595 × 10^4^	2.125 × 10^3^	4.031 × 10^3^	5.954 × 10^2^	2.129 × 10^3^
*Candida albicans*	5.833 × 10^1^	2.041 × 10^2^	3.858 × 10^3^	1.722 × 10^4^	1.240 × 10^1^	3.307 × 10^1^	0.500 × 10^1^	21.325 2.132 × 10^1^
*Alpha-haemolytic streptococcus*	1.420 × 10^2^	3.333 × 10^2^	4.805 × 10^3^	4.978 × 10^3^	1.728 × 10^3^	3.698 × 10^3^	5.686 × 10^3^	2.126 × 10^4^
*Lactobacillus* spp.	6.105 × 10^4^	2.033 × 10^5^	5.426 × 10^3^	1.715 × 10^4^	1.296 × 10^3^	3.291 × 10^3^	9.604 × 10^2^	2.933 × 10^3^
*Corynebacterium* spp.	4.692 × 10^5^	2.040 × 10^6^	4.947 × 10^4^	1.721 × 10^5^	3.012 × 10^3^	4.463 × 10^3^	1.518 × 10^3^	3.466 × 10^3^
*Fusobacterium* spp.	5.512 × 10^3^	2.040 × 10^4^	1.356 × 10^4^	3.226 × 10^4^	4.880 × 10^2^	2.001 × 10^3^	0.454 × 10^1^	2.132 × 10^1^
*Porphyromonas gingivalis*	8.833 ×10^1^	2.815 × 10^2^	2.844 × 10^2^	4.049 × 10^2^	2.252 × 10^2^	3.975 × 10^2^	5.068 × 10^2^	2.130 × 10^3^
*Prevotella intermedia*	1.125 × 10^1^	2.771 × 10^1^	1.847 × 10^2^	3.464 × 10^2^	1.904 × 10^2^	3.631 × 10^2^	6.227 × 10^1^	2.121 × 10^2^
*Veillonella* spp.	1.071 × 10^6^	2.778 × 10^6^	1.877 × 10^7^	3.826 × 10^7^	6.677 × 10^6^	1.988 × 10^7^	2.020 × 10^7^	3.868 × 10^7^
*Corynebacterium* *anaerobium*	0.000	0.000	3.620 × 10^2^	1.719 × 10^3^	1.248 × 10^3^	3.304 × 10^3^	4.153 × 10^2^	1.923 × 10^3^

*^§^* Significant difference between healthy vs. CC-MC; *^η^* Significant difference between MC vs. CC-MC; *^ß^* Significant difference between MC vs. CC-Zr; *^δ^* Significant difference between CC-MC vs. CC-Zr; Post hoc Scheffe. Significant at *p* < 0.05.

## Data Availability

Not available.

## Supplementary Materials

The following are available online at https://www.mdpi.com/article/10.3390/ijms22115463/s1, Table S1: Demographic characteristics and periodontal (PDL) health status of the study participants. Table S2: Multiple comparison of the microbiological composition and counts of the gingival crevicular fluid in all groups before and after treatment.
