# Supplementary material for: Bacteriological Evaluation of Gingival Crevicular Fluid in Teeth Restored Using Fixed Dental Prostheses: An In Vivo Study
Source: Int J Mol Sci. 2021 May 22;22(11):5463. doi: 10.3390/ijms22115463 (PMC8196846; doi:10.3390/ijms22115463)
Supplement: Supplementary file 1 [file ijms-22-05463-s001.zip › ijms-1206002-supplementary.pdf]

International Journal of
Molecular Sciences

MDPI

Table S1. Demographic characteristics and periodontal (PDL) health status of the study participants.

		Control (n-24)	CC_MC (n=35)	CC-Zr (n=35)	MC (n=35)	P-value
**Gender n (%)**	Male	11 (45.8%)	17 (48.6%)	16 (45,75)	17 (48.6%)	P = 0.11
	Female	13 (54.2%)	18 (51.4%)	19 (54.3%)	18 (51.4%)	P = 0.14
**Age (years)**	Range	19-47	18-48	19-49	20-50	P>0,5
	Mean±SD	34.8±7.8	32.3±8.5	35.1±6.8	36.6±8.2	
**PDL status**		Healthy periodontium, no bleeding/periodontal pockets	Clinical examination confirmed the diagnosis of periodontitis in patients			

Table S2. Multiple comparison of the microbiological composition and counts of the gingival crevicular fluid in all groups before and after treatment.

Microorganisms	Healthy vs MC	Healthy vs CC-MC	Healthy vs CC-Zr	MC vs CC-MC	MC vs CC-Zr	CC-MC vs CC-Zr
**Before treatment**						
Enterococcus spp.	0.741	0.976	0.381	0.940	0.883	0.624
Peptostreptococcus spp.	0.980	0.927	0.976	0.993	0.844	0.738
Neisseria spp.	0.979	0.041	0.966	0.065	0.999	0.149
Peptococcus spp.	1	0.683	1	0.553	0.998	0.735
Staphylococcus spp.	1	0.932	0.004	0.923	0.002	0.023
Beta-hemolytic streptococcus	0.362	0.198	1	0.960	0.394	0.221
Candida albicans	0.600	0.977	1	0.846	0.650	0.985
Alpha-haemolytic streptococcus	0.996	0.058	0.808	0.060	0.878	0.400
Lactobacillus spp.	0.995	0.998	0.877	0.970	0.721	0.939
Corynebacterium spp.	0.830	0.015	1	0.078	0.803	0.015
Fusobacterium spp.	0.636	0.901	1	0.636	0.970	0.907
Porphyromonas gingivalis	1	1	0.208	1	0.167	0.207
Prevotella intermedia	1	1	0.285	1	0.229	0.280
**At 6 months of treatment**						
Enterococcus spp.	0.065	0.982	0.992	0.153	0.154	1
Peptostreptococcus spp.	0.653	0.871	0.930	0.189	0.283	0.999
Neisseria spp.	0.735	0.494	0.631	0.961	0.992	0.998
Peptococcus spp.	0.949	0.525	1	0.181	0.958	0.532
Staphylococcus spp.	0.266	0.995	0.657	0.152	0.952	0.497
Beta-hemolytic streptococcus	0.025	1	1	0.024	0.31	1
Candida albicans	0.408	1	1	0.397	0.431	1
Alpha-haemolytic streptococcus	0.968	0.427	1	0.637	0.983	0.492
Lactobacillus spp.	0.833	0.973	0.724	0.980	0.990	0.921
Corynebacterium spp.	0.729	0.980	0.923	0.923	0.333	0.743
Fusobacterium spp.	0.936	0.650	0.589	0.248	0.217	0.999
Porphyromonas gingivalis	0.645	1	0.966	0.692	0.921	0.979
Prevotella intermedia	0.657	0.994	0.271	0.812	0.842	0.395
Veillonella spp.	0.759	0.997	0.271	0.864	0.755	0.362
Corynebacterium anaerobium	0.740	0.571	0.999	0.983	0.825	0.667

At 12 months of treatment						
Enterococcus spp.	0.064	0.997	0.668	0.099	0.631	0.774
Peptostreptococcus spp.	0.318	0.207	0.491	0.207	0.972	0.972
Neisseria spp.	0.927	0.968	0.113	0.999	0.265	0.265
Peptococcus spp.	0.224	0.118	0.913	0.964	0.051	0.025
Staphylococcus spp.	0.150	1	0.957	0.149	0.430	0.961
Beta-hemolytic streptococcus	0.001	0.994	1	0.002	0.002	0.998
Candida albicans	0.555	1	1	0.535	0.565	1
Alpha-haemolytic streptococcus	0.411	0.961	0.346	0.731	0.992	0.630
Lactobacillus spp.	0.213	0.211	0.232	0.999	0.999	1
Corynebacterium spp.	0.463	0.431	0.457	0.998	0.998	1
Fusobacterium spp.	0.556	0.871	0.850	0.37	0.138	1
Porphyromonas gingivalis	0.916	0.975	0.593	0.997	0.890	0.830
Prevotella intermedia	0.159	0.186	0.946	1	0.479	0.497
Veillonella spp.	0.180	0.933	0.198	0.501	0.999	0.493
Corynebacterium anaerobium	0.915	0.155	1	0.370	0.923	0.173

Post hoc Scheffe. Significant at $P<0.05$.
